# Drought, conflict and children’s undernutrition in Ethiopia 2000–2013: a meta-analysis

**DOI:** 10.2471/BLT.16.172700

**Published:** 2017-02-01

**Authors:** Tefera Darge Delbiso, Jose Manuel Rodriguez-Llanes, Anne-Françoise Donneau, Niko Speybroeck, Debarati Guha-Sapir

**Affiliations:** a Center for Research on the Epidemiology of Disasters, Institute of Health and Society, Université catholique de Louvain, Clos Chapelle-aux-Champs 30, 1200 Brussels, Belgium.; b Department of Public Health, University of Liège, Liège, Belgium.; c Institute of Health and Society, Université catholique de Louvain, Clos Chapelle-aux-Champs, Brussels, Belgium.

## Abstract

**Objective:**

To estimate the prevalence of childhood wasting and to investigate the effects of drought and conflict on wasting in crisis-affected areas within Ethiopia.

**Methods:**

We searched the Complex Emergency Database for nutrition surveys carried out in Ethiopia over the period 2000–2013. We extracted data on the prevalence of wasting (weight-for-height *z*-scores below –2) among children aged 6–59 months for areas of Ethiopia that had sufficient data available. Data on any conflict events (irrespective of magnitude or impact) and episodes of seasonal drought affecting the survey areas were extracted from publicly available data sources. Random-effects Bayesian meta-analysis was used to synthesize the evidence from 231 small-scale surveys.

**Findings:**

From the total sample of 175 607 children analysed, the pooled number of children wasted was 21 709. The posterior median prevalence of wasting was 11.0% (95% credible interval, CrI: 10.3–11.7) over the 14-year period. Compared with areas unaffected by drought, the estimated prevalence of wasting was higher in areas affected by moderate levels of drought (posterior odds ratio, OR: 1.34; 95% CrI: 1.05–1.72) but similar in severe drought-affected areas (OR: 0.96; 95% CrI: 0.68–1.35). Although the pooled prevalence of wasting was higher in conflict-affected than unaffected areas, the difference was not plausible (OR: 1.02; 95% CrI: 0.82–1.26).

**Conclusion:**

Despite an overall declining trend, a wasting problem persists among children in Ethiopia. Conflict events did not have a major impact on childhood wasting. Nutrition interventions should go beyond severe drought-prone areas to incorporate areas where moderate droughts occur.

## Résumé

**Objectif:**

Estimer la prévalence de l'émaciation chez les enfants et étudier les effets de la sécheresse et des conflits sur l'émaciation dans les zones touchées par des crises en Éthiopie.

**Méthodes:**

Nous avons recherché dans la base de données Complex Emergency Database des enquêtes sur la nutrition réalisées en Éthiopie sur la période 2000–2013. Nous en avons extrait des données sur la prévalence de l'émaciation (scores z poids/taille –2) parmi les enfants âgés de 6 à 59 mois dans les régions d'Éthiopie qui disposaient de suffisamment de données. Des données sur les conflits (indépendamment de leur ampleur ou de leur impact) et les épisodes de sécheresse saisonnière dans les zones concernées ont été extraites de sources d'informations accessibles au public. Une méta-analyse bayésienne à effets aléatoires a permis de synthétiser les éléments ressortis des 231 enquêtes à petite échelle.

**Résultats:**

Sur un total de 175 607 enfants analysés, le nombre cumulé d'enfants souffrant d'émaciation était de 21 709. La prévalence médiane postérieure de l'émaciation était de 11,0% (intervalle de crédibilité de 95%, ICr: 10,3–11,7) sur cette période de 14 années. Comparée à des zones non affectées par la sécheresse, la prévalence estimée de l'émaciation était plus élevée dans les zones connaissant une sécheresse modérée (rapport des cotes postérieur, RC: 1,34; ICr 95%: 1,05–1,72) mais similaire dans les zones frappées par une forte sécheresse (RC: 0,96; ICr 95%: 0,68–1,35). Même si la prévalence cumulée de l'émaciation était plus élevée dans les zones touchées par des conflits que dans les autres, la différence n'était pas parlante (RC: 1,02; ICr 95%: 0,82–1,26).

**Conclusion:**

Malgré une tendance globalement à la baisse, le problème de l'émaciation des enfants persiste en Éthiopie. Les conflits n'ont pas d'impact majeur sur l'émaciation des enfants. Les interventions en matière de nutrition devraient aller au-delà des zones exposées à de fortes sécheresses pour intégrer celles frappées par des sécheresses modérées.

## Resumen

**Objetivo:**

Estimar la prevalencia de la emaciación en niños e investigar los efectos de las sequías y los conflictos sobre la emaciación en zonas afectadas por la crisis de Etiopía.

**Métodos:**

Se realizaron búsquedas en la Base de Datos sobre Emergencias Complejas en busca de encuestas sobre nutrición realizadas en Etiopía durante el periodo comprendido entre 2000 y 2013. Se extrajeron datos sobre la prevalencia de la emaciación (peso para la talla por debajo de -2 puntuaciones zeta) entre niños de 6 y 59 meses en las zonas de Etiopía con suficientes datos disponibles. Se extrajo información sobre conflictos (independientemente de su magnitud o impacto) y episodios de sequías estacionales que afectaban a las zonas encuestadas de fuentes de datos disponibles públicamente. Se utilizó el metaanálisis bayesiano de efectos aleatorios para sintetizar la evidencia de 231 encuestas a pequeña escala.

**Resultados:**

De la muestra total de 175 607 niños analizados, la cantidad combinada de niños con emaciación fue de 21 709. La prevalencia mediana posterior de emaciación fue del 11,0% (intervalo creíble, ICr, del 95%: 10,3–11,7) durante el periodo de 14 años. En comparación con las áreas no afectadas por la sequía, la prevalencia estimada de la emaciación fue mayor en las zonas afectadas por niveles moderados de sequía (cociente de posibilidades posterior, CP: 1,34; ICr del 95%: 1,05–1,72), pero similar en zonas gravemente afectadas por la sequía (CP: 0,96; ICr del 95%: 0,68–1,35). Aunque la prevalencia combinada de la emaciación fue mayor en zonas afectadas por conflictos que en las que no lo estaban, la diferencia no era plausible (CP: 1,02; ICr del 95%: 0,82–1,26).

**Conclusión:**

A pesar de una tendencia descendente generalizada, los niños de Etiopía siguen sufriendo un problema de emaciación. Los conflictos no supusieron un gran impacto en la emaciación en la niñez. Las intervenciones nutricionales deberían ir más allá de las zonas propensas a sequías e incorporar zonas en la que se produzcan sequías moderadas.

## ملخص

**الهدف:**

تقدير مدى انتشار هزال الطفولة واكتشاف آثار الجفاف والصراع على الهزال في المناطق المتضررة من الأزمة داخل إثيوبيا.

**الطريقة:**

بحثنا في قاعدة بيانات حالات الطوارئ المجمعة لاستطلاعات التغذية التي نُفذت في إثيوبيا خلال الفترة ما بين عامي 2000 و2013. واستخرجنا بيانات حول شيوع الهزال (وكانت المحصلات المعيارية لتناسب الوزن مع الطول أقل من -2‏) بين الأطفال الذين تتراوح أعمارهم ما بين 6 إلى 59 شهرًا في المناطق الإثيوبية التي تتوفر بها بيانات كافية. كما تم استخراج البيانات حول أية صراعات (بغض النظر عن حجمها أو أثرها) ونوبات الجفاف الموسمي التي تؤثر على مناطق الاستطلاع من مصادر البيانات المتاحة للعامة. وتم استخدام التحليل التجميعي وفقًا لنموذج "بايزي" لقياس الآثار الافتراضية العشوائية وذلك لتجميع الأدلة من 231 استطلاعًا على نطاق صغير.

**النتائج:**

من إجمالي العينات التي تم تحليلها وبلغت 175607 أطفال، بلغ إجمالي عدد الأطفال الذين يعانون من الهزال 21709 أطفال. وبلغ متوسط الانتشار اللاحق للهزال نسبة ‏11.0٪ (بهامش موثوقية يبلغ ‏95%، ونسبة ثقة تتراوح ما بين 10.3-11.7) خلال فترة الـ 14 عامًا. وبالمقارنة مع المناطق غير المتضررة من الجفاف، فإن نسبة الانتشار المُقدرة للهزال كانت مرتفعة في المناطق المتضررة من قبل مستويات الجفاف المعتدلة (نسبة أرجحية لاحقة، بنسبة احتمال تبلغ 1.34؛ ونسبة أرجحية مقدارها 95‏‏% تتراوح بين: 1.05-1.72)، لكنها كانت مشابهة في المناطق المتأثرة بالجفاف الشديد (وذلك بنسبة احتمال بلغت 0.96؛ ونسبة أرجحية مقدارها 95‏‏% تتراوح بين: 0.68–1.35). وعلى الرغم من ارتفاع إجمالي انتشار الهزال في المناطق المتضررة من الصراع مقارنةً بالمناطق غير المتضررة، فإن الفارق لم يكن معقولاً (بنسبة احتمال بلغت 1.02؛ ونسبة أرجحية مقدارها ‏95‏% تتراوح بين 0.82–1.26).

**الاستنتاج:**

تظل مشكلة الهزال بين الأطفال في إثيوبيا قائمة على الرغم من الاتجاه العام للانخفاض. و رغم أنه لم يكن للصراعات تأثير كبير على هزال الأطفال، فإنه ينبغي أن تتجاوز عمليات التدخل الغذائية المناطق المعرضة للجفاف وتتجه إلى تغطية المناطق التي تعاني من جفاف معتدل.

## 摘要

**目的:**

旨在评估埃塞俄比亚受危机影响地区的儿童消瘦率和研究这些受危机影响地区干旱与冲突对消瘦的影响。

**方法:**

我们在复杂紧急事件数据库 (Complex Emergency Database) 中检索了 2000 年至 2013 年间在埃塞俄比亚展开的营养调查。并在埃塞俄比亚有充分数据的地区提取了年龄在 6 至 59 个月的儿童的消瘦率数据（即，体重别身高 *z* 评分低于 –2）。我们从公开性数据来源中提取了影响所调查地区的任何冲突事件（不考虑量级或影响）和季节性干旱数据。并且采用随机效应的贝叶斯元分析法汇总了 231 项小规模调查中的证据。

**结果:**

在接受分析的全部 175 607 例儿童中，汇总的消瘦儿童数为 21 709 例。后者在 14 年间的消瘦率中值为 11.0%（95% 置信区间，CrI：10.3–11.7）。与未受干旱影响地区相比，受中度干旱影响地区的消瘦率估计值较高（后者的比值比 OR 为：1.34; 95% CrI: 1.05–1.72），但类似于受重度干旱影响地区 (OR: 0.96; 95% CrI: 0.68–1.35). 尽管受冲突影响地区的汇总消瘦率高于未受影响地区，但该区别似乎并不合理 (OR: 1.02; 95% CrI: 0.82–1.26)。

**结论:**

尽管整体呈减弱趋势，但消瘦问题在埃塞俄比亚儿童间依旧存在。冲突事件并不是儿童消瘦的主要影响因素。营养干涉不应局限于重度干旱频发地区，还应纳入中度干旱地区。

## Резюме

**Цель:**

Определить распространенность гипотрофии у детей и изучить влияние засухи и конфликтов на гипотрофию в затронутых кризисом областях Эфиопии.

**Методы:**

Авторы выполнили поиск исследований в области питания, проведенных в Эфиопии в период между 2000 и 2013 годами, в базе данных по сложным чрезвычайным ситуациям. Были получены данные по распространенности гипотрофии (*z*-показатели соотношения веса и роста менее −2) среди детей в возрасте 6–59 месяцев для тех областей Эфиопии, для которых было доступно достаточно данных. Данные обо всех случаях конфликтов (независимо от значительности или влияния) и эпизодах сезонной засухи, оказывающих воздействие на исследуемые области, были получены из общедоступных источников. С помощью байесовского метаанализа с использованием модели случайных эффектов были обобщены фактические данные из 231 малоформатного исследования.

**Результаты:**

Из общей выборки, которая включала 175 607 детей, участвовавших в анализе, суммарное количество детей с гипотрофией составило 21 709. Апостериорная медианная распространенность гипотрофии составила 11,0% (95%-й доверительный интервал, ДИ: 10,3–11,7) за 14-летний период. По сравнению с областями, не подвергшимися влиянию засухи, подсчитанная распространенность гипотрофии была выше в областях, затронутых засухой в умеренной степени (апостериорное отношение шансов, ОШ: 1,34; 95%-й ДИ: 1,05–1,72), и такой же в областях, сильно затронутых засухой (ОШ: 0,96; 95%-й ДИ: 0,68–1,35). Хотя совокупная распространенность гипотрофии в областях, затронутых конфликтом, была выше, чем в незатронутых областях, разница не была достоверной (ОШ: 1,02; 95%-й ДИ: 0,82–1,26).

**Вывод:**

Несмотря на общую тенденцию к снижению, дети в Эфиопии по-прежнему сталкиваются с проблемой гипотрофии. Случаи конфликтов не оказывали существенного влияния на гипотрофию у детей. Охват вмешательств в области питания следует расширить за пределы областей, подверженных сильной засухе, с включением областей, где случаются умеренные засухи.

## Introduction

Since 1990, considerable progress has been made towards improving child health in the world.^1^^,^^2^ Nonetheless, worldwide 50 million children younger than five years had acute malnutrition in 2014^1^ and nearly 6 million children died in 2015.^2^ The burden is particularly heavy in Africa, where conflict, political fragility and drought are more prevalent.^3^ These events affect food security and nutrition by limiting food accessibility, impacting health services and disturbing the care structure within the society.^4^^,^^5^ Several studies have documented the negative effect of conflict^5^^–^^10^ and drought^7^^,^^11^ on child health and nutrition.

Ethiopia has been affected by drought and starvation on a large scale since the mid-1980s. Untimely, abnormally low and infrequent rainfall has been increasing the frequency and impact of droughts in recent years.^12^ Drought is the most complex and detrimental natural hazard and has a substantial impact in countries such as Ethiopia where the economy and livelihood are predominantly dependent on subsistence rain-fed agriculture.^12^ The country has also experienced several conflicts, both within the country and with neighbouring states such as Eritrea and Somalia.^13^ Nevertheless, in the decade 2000–2011, the country showed improvements in key economic and development indicators^14^^,^^15^ and in 2014 Ethiopia was on track to achieve six of the eight millennium development goals (MDGs).^16^ Yet child undernutrition is still a concern, with an estimated 4 819 770 (40%) of the child population of 12 049 424 being stunted and about 1 084 448 (9%) being wasted in 2014.^17^ According to the Cost of Hunger in Africa study,^18^ undernutrition in Ethiopia was responsible for an estimated 378 000 child deaths in 2005–2009 and cost about 16.5% of the country’s gross domestic product (GDP), an estimated 4.7 billion United States dollars, in 2009 alone. The problem of undernutrition is worse in crisis-affected areas within the country, where food insecurity is heightened due to climate shocks and conflicts.^10^^,^^19^

Although several small-scale surveys have been conducted by humanitarian organizations in crisis-affected areas, we only found one study which investigated the associations among child undernutrition, conflict and variability in the general ecosystem in East Africa including Ethiopia.^9^ Wasting reflects recent weight loss and has been shown to be a good predictor of child mortality.^20^ Consequently, it is a preferred index of nutritional status in humanitarian emergencies and a proxy indicator for the general health and welfare of the entire population.^5^^,^^21^ Thus, examining the effects of drought and conflict on the prevalence of wasting in children would provide knowledge to guide intervention strategies. Moreover, generated estimates could be a useful baseline for future surveys in crisis-affected areas. We sought to provide summary estimates of the prevalence of wasting among children aged 6–59 months and investigate the effects of drought and conflict on the prevalence of childhood wasting in regions of Ethiopia that had sufficient data available.

## Methods

### Nutrition data

**Fig. 1:**
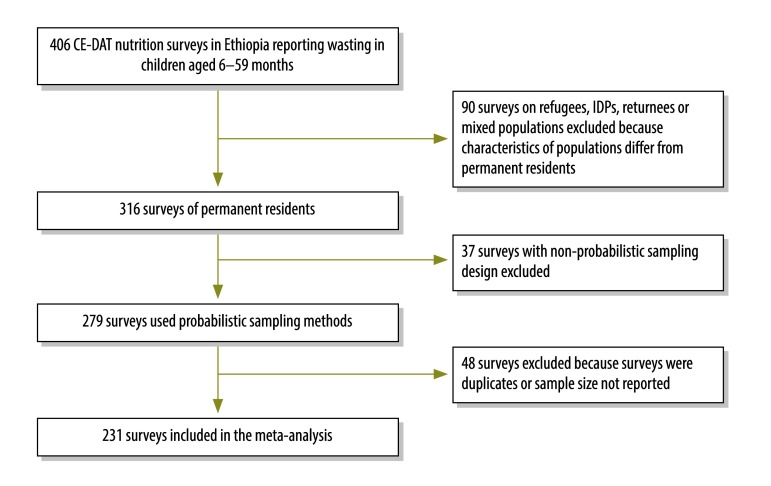
Flowchart of small-scale surveys in Ethiopia (2000–2013) included in the meta-analysis

CE-DAT: Complex Emergency Database; IDP: Internally Displaced Person.

We obtained all data from publicly available databases (Table 1). First, we searched the Complex Emergency Database (CE-DAT) for all nutrition surveys carried out in Ethiopia from 1 January 2000 to 31 December 2013. CE-DAT compiles data on commonly used health indicators (e.g. nutrition, vaccination and mortality) from small-scale surveys conducted in areas affected by complex emergencies.^22^^,^^23^ The Emergency Nutrition Coordination Unit of Ethiopia, a government department, leads the coordination and quality assurance of the surveys in the field,^28^ while the CE-DAT team validates the surveys before entering the results in the database.^22^^,^^23^ From 406 small-scale nutrition surveys identified we excluded duplicate studies; studies with non-probabilistic sampling design or unreported sample size; and studies of refugees, internally displaced persons, returnees (displaced persons who have returned to their place of origin or habitual residence) or mixed populations (refugees, internally displaced persons and residents living together). Based on our inclusion criteria, we used data from 231 surveys (Fig. 1).

**Table 1:** Key characteristics of the databases used in the analysis of the effects of drought and conflict on the prevalence of wasting in crisis-affected areas within Ethiopia, 2000–2013

Variable	Database			
	Complex Emergency Database^22^^,^^23^	SPEI Global Drought Monitoring^24^^,^^25^	Armed Conflict Location & Event Project^26^	Uppsala Conflict Data Program^27^
Website	http://cedat.be/	http://sac.csic.es/spei/	http://www.acleddata.com/	http://ucdp.uu.se/
Data compiled	Nutrition (e.g. childhood wasting and stunting), mortality and vaccination indicators from areas affected by complex emergencies	Intensity, magnitude, duration and spatial extent of drought episodes	Political violence event data, disaggregated by time, agent, type and space	Event-based georeferenced data set on organized violence
Time coverage	2000–2013	1950– ongoing	1997– ongoing	1946– ongoing
Spatial coverage	50+ developing countries	Global	60+ countries in Africa and Asia	35+ countries in Africa and East Asia
Sample	3309 surveys	SPEI of the globe at a spatial resolution of 0.5 degrees	100 000+ conflict events (October 2015)	21 860+ violent events (October 2015)
Data sources	Surveys from United Nations agencies; country clusters; academic institutions; NGOs; ministries of health; and published peer-reviewed journals	Calculated based on climatic water balance using monthly precipitation and temperature data	Media reports; humanitarian agency and NGO reports	Media and research reports; international and multinational agency and NGO documents; and regional experts

NGO: nongovernmental organization; SPEI: standardized precipitation evapotranspiration index.

From these surveys, we extracted data on the prevalence of wasting. Wasting was calculated from weight and height measurements of children aged 6–59 months to produce weight-for-height *z*-scores. A *z*-score index below −2 is a sign of acute undernutrition and is defined as wasting.^29^ The prevalence of wasting in the surveys was reported in relation to the United States National Center for Health Statistics (NCHS) reference population or the 2006 World Health Organization (WHO) standard. To obtain comparable data over time, we converted estimates based on the NCHS standard into the WHO standard using a conversion algorithm.^30^

For each survey, we also extracted data on sample size, the date when the survey commenced, the administrative area name and the GPS (geographic coordinate system) coordinates of the study location.

### Conflict events data

We compiled data on any conflict events affecting each survey location. We used data from two sources: the Armed Conflict Location and Event Data Project (ACLED) and the Uppsala Conflict Data Program (UCDP) Armed Conflict Dataset (Table 1). ACLED contains georeferenced data on political violence and protests,^26^ while UCDP provides information on armed conflicts in which the use of armed force between two parties (at least one of which is the government of a state) results in at least 25 battle-related deaths in a year.^27^ Both ACLED and UCDP data are assembled from various primary and secondary sources (e.g. online databases, print media and reports from nongovernmental organizations [NGOs] and regional experts) and then aggregated, coded and cross-checked by experienced researchers to assure consistency, accuracy and compatibility with the inclusion criteria.^26^^,^^27^ We combined the two data sets, to capture all conflict events occurring over the study period. We considered conflict events irrespective of their magnitude or impact, to accommodate smaller conflicts that might have an impact on the livelihood of the population. We created a binary variable to indicate the occurrence of a conflict event (yes/no) in a given location within six months before the survey starting date. Finally, we extracted the specific locations of the conflict events and matched them with the wasting data from CE-DAT in the same GPS location as each survey.

### Drought episodes data

We also compiled data on episodes of drought affecting each survey location. We extracted the data from the Global Drought Monitor database. The database provides real-time information about drought conditions at the global scale with a spatial resolution of 0.5 degrees (about 50 km) and a monthly time resolution (Table 1). We used the standardized precipitation evapotranspiration index, which is a multi-scalar index used to quantify the precipitation deficit over time and space for different timescales. The index enables identification of the severity of droughts, which is vital information for assessing different responses needed to drought.^24^^,^^25^ We used the three-month index, which is a short-term or seasonal drought index,^25^ as wasting is mainly the result of sudden or recent weight loss due to short-term shocks such as drought. Moreover, the three-month index better captures drought events affecting agriculture.^24^^,^^25^ Thus, the short-term effect of drought on childhood wasting over the preceding three months of a survey could be adequately captured. Positive index values indicate a wet period while negative values indicate a dry period.^25^ We categorized the index values as: no drought (value > 0), mild drought (−1 < value ≤ 0), moderate drought (−1.5 < value ≤ −1) and severe to extreme drought (value ≤ −1.5).^31^ The drought episodes at specific locations in a given period were matched with the wasting data from CE-DAT in the same month and GPS location as each survey.

### Statistical analysis

We identified and measured heterogeneity between the surveys using two statistical methods: the between-survey variance (*tau* statistic, *τ^2^*) and the percentage of variability in effect size due to heterogeneity (*I^2^*).^32^ We then fitted a random effects meta-analysis to address the heterogeneity. To explain the heterogeneity, we further fitted meta-analysis of subgroups and random-effects meta-regression by including study-level covariates (moderators): conflict events, drought episodes, survey years and regions. We also tested the interaction effect to assess whether the association between drought and wasting varied by conflict status.

We used a Bayesian approach for meta-analysis to accommodate all parameter uncertainties in the model and to borrow strength from other surveys to improve parameter estimation.^33^ As we had compiled a large number of surveys, each of which had a substantial sample size, we wanted our posterior inference to reflect the information in our data set without being influenced by the choice of prior distribution. Consequently, we assigned non-informative prior distributions for the model parameters.

The number of wasted children, r*_i_* (*i *= 1, …, 231),**was modelled using a binomial distribution: *r_i_ ~ binomial *(* p_i_ , n_i _*), where *p_i_* is the prevalence of wasting in survey *i* and considered as a random variable and *n_i_* is the sample size of survey *i*. The logit-transformed prevalence was assumed to follow a normal distribution: logit**(*p_i_*) = *µ*_i_ and *µ*_i_ *~ normal *(*µ, τ^2^*), where *µ* was the estimated common prevalence and *τ^2^* was the between-survey variance. The meta-regression model was then formulated by extending the above model to include moderators (*x_i_*) and is given by logit**(*p_i_*) = *µ*_i_ + *βx_i_* , where *β* is the slope coefficient. Then non-informative priors were assigned to the parameters: *µ* *~ normal* (0,10^6^), *β ~normal *(0,10^6^) and *τ ~ gamma *(0.001, 0.001). We ran the chain for 100 000 iterations with a burn-in length of 10 000 iterations to ensure convergence of Markov chain. The convergence was assessed by visual inspection of the trace plots and autocorrelation plots.^34^

We report the posterior median estimates of the prevalence of wasting as percentages and the odds ratios (OR) with 95% credible intervals (CrI). Results were judged statistically significant if the 95% CrI did not overlap 1.0.

The statistical analyses were conducted using WinBugs software version 14 (MRC Biostatistics Unit, Cambridge, United Kingdom of Great Britain and Northern Ireland). We used the metafor package for R statistical software (R Project for Statistical Computing, Vienna, Austria) for constructing the forest plots.

## Results

### Survey descriptions

**Fig. 2:**
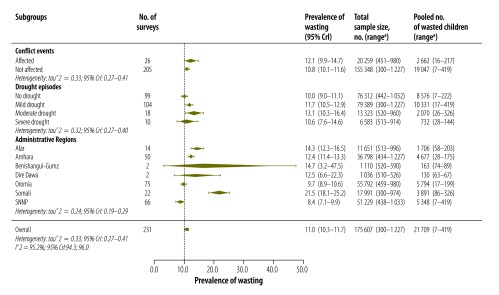
Summary of the pooled prevalence of wasting among children aged 6–59 months from 231 surveys in crisis-affected areas within Ethiopia, 2000–2013

Fig. 2 presents a summary of the survey characteristics and pooled prevalence of wasting among 175 607 children aged 6–59 months from 231 surveys in crisis-affected areas within Ethiopia between 2000 and 2013. The sample sizes ranged from 300–1227 children per survey. Further details of the surveys are available from the corresponding author.

CrI: credible intervals: SNNP: Southern Nations, Nationalities, and People’s.

^a ^Across surveys.

Notes: Wasting was defined as weight-for-height z-scores below −2, standardized to WHO reference.^30^ Prevalence was the posterior median estimates of wasting, expressed as percentages of the number of children in each category, with their 95% CrI. Conflict events were occurrence of a conflict in a given location within six months of the survey’s starting date, irrespective of magnitude or impact. Data from the Armed Conflict Location and Event Project^26^ and the Uppsala Conflict Data Program Armed Conflict Dataset^27^ were combined into a binary variable. Drought episodes in a given location were based on the three-month standardized precipitation evapotranspiration index (SPEI) from the Global Drought Monitor database:^25^ no drought (SPEI > 0), mild drought (−1 < SPEI ≤ 0), moderate drought (−1.5 < SPEI ≤ −1) and severe to extreme drought (SPEI ≤ −1.5).

The dates of the surveys were distributed as follows: 67 (29.0%) were conducted between 2000 and 2004, 97 (42.0%) were between 2005 and 2009 and 67 (29.0%) between 2010 and 2013. The surveys covered seven of the 11 Ethiopian regions. Most of the surveys were from three administrative regions: Oromia (75; 32.5%), Southern Nations, Nationalities, and People’s (66; 28.6%) and Amhara (50; 21.6%). 

We ascertained that 26 (11.3%) of the surveys, involving a pooled number of 20 259 children, were conducted in areas affected by some kind of conflict in the six months before the surveys; 155 348 children lived in areas where no conflicts were recorded. Overall, 132 (57.1%) of the surveys were from areas affected by mild to extreme drought three months before the survey implementation month. The pooled numbers of children affected by mild drought were 79 389, moderate drought 13 323 and severe drought 6583; a total of 76 312 children were living in areas where no episodes of drought were recorded.

### Pooled prevalence

**Fig. 3:**
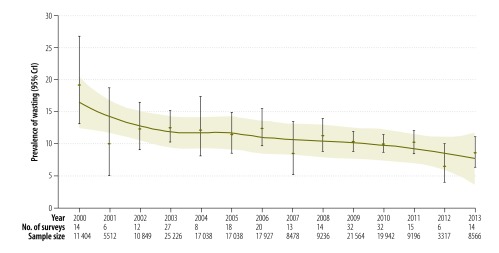
Trends in the prevalence of wasting among children aged 6–59 months from 231 surveys in crisis-affected areas within Ethiopia, 2000–2013

The heterogeneity statistic showed a large variability across the surveys compiled (*τ^2^* = 0.33; *I^2^* = 95.2%). Overall, a total of 21 709 children were affected by wasting and the estimated pooled prevalence of wasting from the 231 surveys was 11.0% (95% CrI: 10.3–11.7). Moreover, 143 of the surveys (61.9%) reported a wasting prevalence of over 10%. The highest regional prevalence was observed in Somali region (21.5%), where a total of 3891 children were affected by wasting, and the lowest was in Southern Nations, Nationalities, and People’s region (8.4%; 5348 children; Fig. 2). The estimated prevalence of wasting decreased steadily over the study period from 19.1% (2233 children) in 2000 to 8.5% (817 children) in 2013 (Fig. 3).

CrI: credible intervals

Notes: Prevalence was the posterior median estimates of wasting, expressed as percentages of the number of children in each year with their 95% CrI. The smoothed line is the local polynomial regression fit and the shaded region is its 95% confidence interval. Wasting was defined as weight-for-height z-scores below −2, standardized to WHO criteria.^30^

The pooled prevalence of wasting was estimated to be 13.1% among children living in areas affected by moderate drought (a pooled total of 2070 children; Fig. 2) and this was higher than in areas with no drought (10.0%; 8576 children). In areas affected by severe drought the estimated prevalence of wasting was similar (10.6%; 732 children) to that in areas unaffected by drought. 

The estimated prevalence of wasting was 12.1% (a pooled total of 2662 children) in conflict-affected areas and 10.8% in areas unaffected by conflict (19 047 children).

### Meta-regression analysis

In survey areas affected by moderate drought the odds of the prevalence of wasting were higher than in areas unaffected by drought (OR: 1.34; 95% CrI: 1.05–1.72; Table 2), whereas in areas affected by severe drought there was no difference compared with unaffected areas (OR: 0.96; 95% CrI: 0.68–1.35). Conflict in the survey area had no plausible effect on the odds of the prevalence of wasting (OR: 1.02; CrI: 0.82–1.26). A one-year increase in the survey period reduced the odds of the prevalence of wasting by 4% (OR: 0.96; 95% CrI: 0.94–0.98) (Table 2). We reported the model without interaction, as the interaction between drought and conflict was found to be implausible.

**Table 2:** Results of meta-regression from 231 surveys of the prevalence of wasting among children aged 6–59 months in crisis-affected areas within Ethiopia, 2000–2013

Category	Posterior OR (95% CrI)
**Conflict events affecting survey area^a^**	
No	Ref.
Yes	1.02 (0.82–1.26)
**Drought episodes affecting survey area^b^**	
None	Ref.
Mild	1.04 (0.91–1.19)
Moderate	1.34 (1.05–1.72)
Severe	0.96 (0.68–1.35)
**Survey period^c^**	0.96 (0.94–0.98)
**Administrative region of survey**	
Afar	1.51 (1.15–2.00)
Amhara	1.29 (1.08–1.54)
Benishangul-Gumz	1.61 (0.80–3.26)
Dire Dawa	1.53 (0.75–3.13)
Oromia	Ref.
Somali	2.21 (1.74–2.81)
Southern Nations, Nationalities, and People’s	0.82 (0.69–0.97)

OR: odds ratio; CrI: credible interval; Ref.: reference group.

^a^ Conflict events were occurrence of a conflict in a given location within six months of the survey starting date, irrespective of magnitude or impact.

^b^ Drought episodes in a given location were based on the three-month standardized precipitation evapotranspiration index (SPEI): no drought (SPEI > 0), mild drought (−1 < SPEI ≤ 0), moderate drought (−1.5 < SPEI ≤ −1) and severe to extreme drought (SPEI ≤ −1.5).

**^c^** For a one-year increase in the survey period.

Notes: Data were estimated from a pooled sample of 175 607 children. See Fig. 2 for the number of surveys, samples sizes, numbers of children affected and prevalence of wasting by category. Wasting was defined as weight-for-height z-scores below −2.

## Discussion

Here we present estimates of the prevalence of childhood wasting in crisis-affected areas within Ethiopia, using pooled data from 231 small-scale surveys. We used a Bayesian approach to meta-analysis to improve estimates by taking into account all parameter uncertainties and borrowing strength from all available surveys. Overall, the pooled prevalence of wasting was 11.0% and showed a decreasing trend over the 14-year period covered in the meta-analysis. In nearly 62% of the surveys the prevalence of wasting exceeded WHO’s 10% threshold for an emergency response, an indication of a serious concern for child nutrition.^21^ In a meta-regression analysis we also showed that moderate drought-affected areas had a higher prevalence of wasting than areas unaffected by drought. Conflict in an area, however, was not associated with wasting.

Three results are worth highlighting. First, the prevalence of wasting declined over the period of the study. Economic growth in a country alleviates poverty, thereby reducing the risk of hunger and malnutrition.^4^ In the period 2000–2011 Ethiopia’s average GDP grew by over 10% annually^14^ and the population living below the national poverty line declined from 44% in 2000 to 30% in 2011.^15^ Therefore the population below the minimum dietary energy consumption dropped from 41.9% in 2000 to 33.6% in 2011.^16^ The country achieved the MDG target of reducing extreme hunger by half.^4^ Ethiopia launched a national nutrition strategy in 2008 with the aim of improving food and nutrition security.^35^ The different programmes that are in place to improve nutrition practices^17^ – including health education, provision of micronutrients and treatment of malnourished children – could explain the declining trend of childhood wasting found in our study. Despite these improvements in development and a commitment and investment in nutrition interventions in Ethiopia, we found that the prevalence of wasting in crisis-affected areas was high. In particular, Somali, Afar and Amhara regions had higher prevalence than other areas. This could be due to the higher food insecurity problems in parts of the regions as a result of recurrent droughts and land degradation.^19^

Second, although the link between drought and nutrition is well recognized, it is often indirect and complex. The quality and quantity of nutrient intake is affected by reduced food production as a result of drought.^11^ Our results showed that the prevalence of wasting was 34% higher in areas affected by moderate drought relative to non-affected areas, whereas no such difference was observed in areas affected by severe drought. In 2005, the Ethiopian government, in collaboration with partner organizations, launched the Productive Safety Net Programme. The programme aimed to ensure food supplies to chronically food-insecure areas that are highly vulnerable to climate shocks.^36^ Hence, areas affected by severe drought are more likely to benefit from the programme and this might explain why no effect on wasting was seen in these areas. Furthermore, the country’s early warning system,^37^ which targets the areas affected by severe drought, may also help mitigate the impact of severe droughts. Our results underscore the need for interventions to go beyond predefined severe drought-prone areas, with strengthening of early warning systems and intervention programmes to benefit people living in moderate drought-prone areas too. On the other hand, if we hypothesize that there is a potentially high mortality among children in areas affected by severe drought, survival bias might explain the similar prevalence of wasting in severe drought-affected areas and unaffected areas. This assumption is corroborated by a study that found drought-affected areas experienced higher child mortality in the 2002–2003 drought in Ethiopia.^38^ Unfortunately, the mortality and cause of death data in the studied surveys were not detailed enough to test our assumptions and to provide a more holistic view of the drought impact on mortality.

Third, conflict and a high prevalence of acute undernutrition are reported to be associated.^39^ Conflict triggers food insecurity and affects nutrition through disrupting crop production; destroying food stores and livestock; forcing people to eat food with lower nutritional value; affecting market food supply and price inflation; pushing people to live in unhealthy environments; impacting health services; and affecting productivity and family care structures.^4^^,^^5^^,^^40^ However, in our meta-analysis, we found no evidence of a difference between the prevalence of wasting in areas affected and unaffected by conflict. The effect of conflict on livelihood and nutrition depends on the nature, magnitude and duration of the conflict.^40^ In our study, most of the conflict events which occurred during the specified time period were short-lived (mostly one day) and of low magnitude (mainly no fatalities). They may therefore not have had a major impact on the nutrition situation of the community in general and children in particular. Our finding is supported by previous studies,^9^^,^^41^ which found no association between the prevalence of wasting and exposure to conflict events.

Our study had limitations. First, we compiled surveys that were conducted by different organizations for practical and operational purposes; hence the survey results are rarely peer-reviewed, and sharing or publication bias cannot be ruled out.^22^^,^^23^ However, most of the surveys used standardized methods of nutrition surveys such as the SMART (Standardized Monitoring and Assessment of Relief and Transitions) method.^42^ Furthermore, CE-DAT is a unique and well-established data source for nutrition and mortality indicators in humanitarian emergencies.^22^^,^^23^ Second, while the assessment of CE-DAT coverage is a future area for improvement of the database,^22^^,^^23^ the surveys are representative of small geographical areas, a key characteristic to ascertain the small-scale impact of drought and conflict on nutrition. Third, potential underlying determinants (e.g. coping strategies, diseases and food and non-food interventions)^39^ which could explain part of the observed variability are not available in CE-DAT. Fourth, the locations for conducting the surveys were likely influenced by suspicions of higher levels of undernutrition. However, our aim here was not to assess malnutrition levels in the country but in crisis-affected areas within Ethiopia. Fifth, the ACLED and UCDP databases mainly collect conflict event data from the media, online journals and nongovernmental organizations’ publications.^26^^,^^27^ These could introduce reporting bias (selective reporting or omissions) for political or other reasons. To reduce the bias, we combined the two databases and also considered only the occurrence of conflict, as data on the impact of conflicts (e.g. deaths) are more likely to suffer from reporting bias from different organizations. Although these limitations constrain our study’s ability to fully explain the links between drought or conflict and nutrition, the findings still provide evidence to support policies and programmes designed to mitigate the health effects of drought and conflict.

In conclusion, the pooled prevalence indicates a persistent childhood wasting problem in Ethiopia. Therefore, national nutrition strategies should target areas of persistent undernutrition. Although areas affected by severe drought must remain a national priority for specific targeted actions, nutrition policy should consider interventions that go beyond the predefined, severe drought-prone areas and incorporate areas where moderate droughts occur.
